# Bee Inspired Novel Optimization Algorithm and Mathematical Model for Effective and Efficient Route Planning in Railway System

**DOI:** 10.1371/journal.pone.0166064

**Published:** 2016-12-08

**Authors:** Kah Huo Leong, Hamzah Abdul-Rahman, Chen Wang, Chiu Chuen Onn, Siaw-Chuing Loo

**Affiliations:** 1 Faculty of Science Technology Engineering and Mathematics (STEM), International University of Malaya-Wales, Kuala Lumpur, Malaysia; 2 College of Civil Engineering, Huaqiao University, 361021, Xiamen, China; 3 Department of Civil Engineering, Faculty of Engineering, University of Malaya, Kuala Lumpur, Malaysia; 4 Centre of Building, Construction & Tropical Architecture, Faculty of Built Environment, University of Malaya, Kuala Lumpur, Malaysia; Lanzhou University of Technology, CHINA

## Abstract

Railway and metro transport systems (RS) are becoming one of the popular choices of transportation among people, especially those who live in urban cities. Urbanization and increasing population due to rapid development of economy in many cities are leading to a bigger demand for urban rail transit. Despite being a popular variant of Traveling Salesman Problem (TSP), it appears that the universal formula or techniques to solve the problem are yet to be found. This paper aims to develop an optimization algorithm for optimum route selection to multiple destinations in RS before returning to the starting point. Bee foraging behaviour is examined to generate a reliable algorithm in railway TSP. The algorithm is then verified by comparing the results with the exact solutions in 10 test cases, and a numerical case study is designed to demonstrate the application with large size sample. It is tested to be efficient and effective in railway route planning as the tour can be completed within a certain period of time by using minimal resources. The findings further support the reliability of the algorithm and capability to solve the problems with different complexity. This algorithm can be used as a method to assist business practitioners making better decision in route planning.

## Introduction

Railway system (RS) is growing in popularity for major cities around the world as it provides significant transit capacity and become an essential infrastructure that is needed to serve growing transportation demands. One of the main reasons RS is developed in the city is to reduce the traveling cost but due to expansion of the transit networks and structures, often, unplanned travel will cause unnecessary waste of time and passengers congestion in the stations [1]. Transit planning can be an extremely complex due to multiple variables that will affect the quality of the solution [2, 3]. For instance, departure time, train intervals, operating characteristics, minimal and maximum headway of lines. There are many optimization algorithms to solve routing problems evolved such as ant colony optimization, greedy algorithm, genetic algorithm, termite colony algorithm and bat algorithm but very limited literature is found related to Railway Traveling Salesman Problem (RTSP) [4].

**Fig 1:**
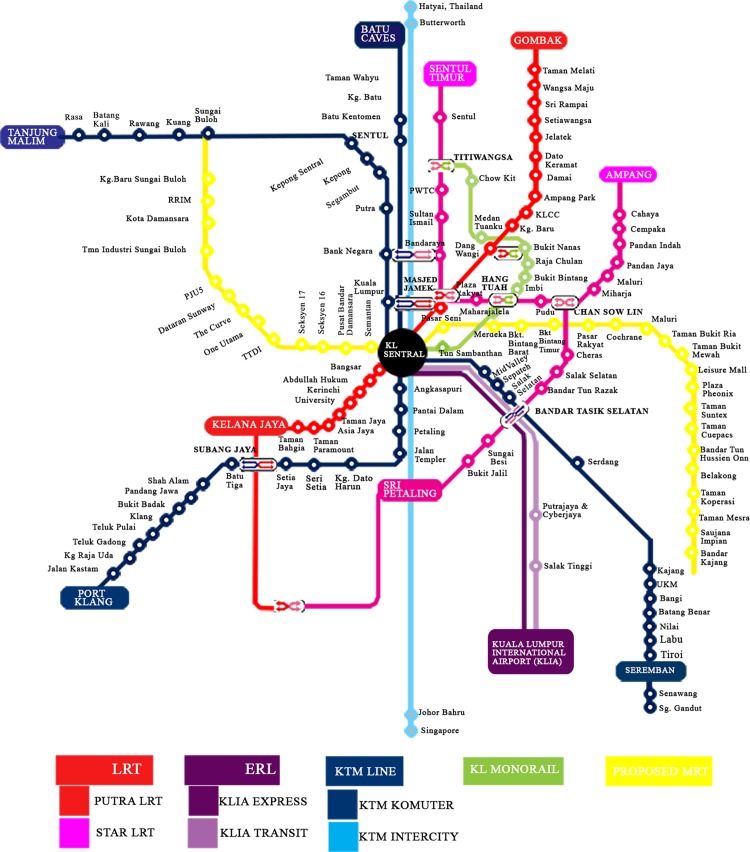
Malaysia railway system map (for illustrative purposes only).

In Malaysia, railway transport is one of the most commonly used public transportation by many Malaysians, business practitioners and travellers to travel from one destination to another daily. LRT, KL Monorail, Airport Express Rail Link and KTM Commuter are the lines that connect in the Malaysian RS (Fig 1). According to Brenda Ch’ng [5], RS in Malaysia has a daily ridership of more than half a million in year 2014 and the number is expected to doublerf when new lines are ready in the future. People tend to use this mode of transport to travel in the city in order to save time and costs by avoiding traffic congestion, spending time looking for parking bay and traveling on toll roads. When the RS network is expanded, choosing the shortest route to multiple destinations will be difficult due to the complexity of the network design and structure.

## Background

### Traveling Salesman Problem (TSP)

TSP is a mathematical problem introduced by Sir William Rowan Hamilton and Thomas Penyngton Kirkman in 18^th^ century and later promoted by Hassler, Whitney & Merrill at Princeton [6]. TSP is to find the shortest possible route that enable the traveller visits each city exactly once and returns to the origin city [7]. There is a growing body of literature that recognises the importance of TSP and it is not only studied by mathematicians and computer scientists but researchers from other industries to find out how TSP can help in operations planning and decision making [8]. TSP is one of the most well-known routing problems that researchers are still looking for the best solutions yet [9]. In TSP, a salesman was given a set of cities along with the travel cost between these cities. The salesman’s task is to complete a tour by visiting all of those cities exactly once and then return to the point where the salesman started his journey. The primary objective is to find the best possible solution with the minimum cost to travel [10].

Solving TSP by using exhaustive search methods is possible but not practical because it will be very costly to generate solutions when possible routes exponentially increased. Due to this reason, no efficient solution to the general case of TSP (for all variants) has been found yet [9]. Route planning in railway systems is one of the variants of TSP. This suggests that the route planning in railway system requires a different set of variables in order to develop a technique or algorithm to solve the TSP for railway system. Unfortunately, the technique or algorithm to solve TSP for railway system is yet available in the existing body of knowledge. Therefore, no current TSP technique or algorithm can be applied in solving the route planning in railway systems. Therefore, this study attempts to bridge the gap by developing a TSP solution for route planning in railway system. In spite of the computational difficulty of the problem, various known techniques have been introduced by researchers to generate the best solution to the problem. These techniques can be classified into 2 categories that are exact and approximation algorithms [11].

### Swarm Intelligence (SI)

SI is known as an efficient meta-heuristic approximation algorithm used by researchers to solve TSP and optimization related problems [12]. SI was introduced in 1989 by Beni and Wang [13] and later defined by Bonabeau as the emergent collective intelligence of groups [12]. It is broadly defined as a group of individuals acting collectively in ways that seem intelligent and often inspired from natural or artificial process [7]. It constituted a swarm of simple agents interacts locally with each other and environment to discover the unknown knowledge [14]. Examples of swarm intelligence based models are ant colony optimization, particle swarm optimization, bee colony optimization and artificial immune system. A modified bee colony optimization algorithm is proposed in this paper to solve Railway Traveling Salesman Problem (RTSP).

#### Bee algorithm as approximation method

Meta-heuristic and approximation method are defined as the upper-level general methodologies and it can be used as a guidance of strategies to design underlying heuristics to solve specific optimization problems [15]. Intensification and diversification are the two important characteristics of meta-heuristic methods. Intensification is selecting the best candidates from the best solutions gathered and diversification is making sure that the algorithm works efficiently to explore the search space randomly [16].

Bee inspired algorithm such as Bee Colony Optimization (BCO) has been successfully used to solve many problems in engineering, operations and management related fields [17]. The general idea of BCO is constructing multi agent system that consists of artificial bees in a colony, where they will find the best solution during the process of collecting nectars. Bee behaviour in nature has inspired researchers to design various algorithms and solutions to solve difficult combinatorial optimization problems such as TSP [18]. Although various social insect species based algorithms have successfully solved various complex problems, Teodorovic [18] claimed that bee behaviour in nature has inspired more significant solutions to the problems. According to Aghazadeh and Meybodi [19], bees will adapt their behaviour according to the environment to accomplish a task by using collective intelligence. Basically, all the insect colonies have their own division of work based on their system and this applies to bee colonies as well.

For instance, honeybee colony is distributed in multiple directions for long distances at a same time in order to find more food sources [20]. The deployment of its foragers to better fields is the success criteria of the bee colony. The bee colony follows the rules that if the flower was patched with plenty amount of nectar then the flower will be visited by more bees and vice versa. Baykasoglu, Ozbakir and Tapkan [21] identified food and foragers are the two important criteria in a bee system (Table 1).

**Table 1:** Types of foragers in bee systems.

Types of foragers	Description
**Unemployed bees**	The bee initializes its search as an unemployed forager if those bees have zero knowledge about the food sources in the search field. The unemployed forager can be divided into two groups which are:
	**Scout Bee:** The scout bee is decided if the bee starts searching spontaneously without any knowledge. The percentage of scout bees varies from 5% to 30% according to the information into the nest. The mean number of scouts averaged over conditions is about 10%.
	**Recruit:** The bee will start searching if the unemployed forager attends to a waggle dance present by some other bee by using the knowledge from waggle dance.
**Employed bees**	Employed forager memorizes the location of the food source is raised from the new recruit bee that finds and exploits the food source. After a portion of nectar from the food source was loaded from the employed foraging bee, the nectar will be unloaded to the food area in the hive after the bee return to the hive. The residual amount of nectar for the foraging bee is depending on three possible actions:
	• Foraging bee abandons the food source and become an unemployed if the nectar amount decreased to a low level or exhausted.
	• Employed foragers can continue to forage without sharing the food source information with the nest mates if sufficient amount of nectar in the food source.
	• Inform other nest mates about the food source by going to the dance area to perform waggle dance.
**Experienced foragers / recruiters**	Experienced foragers use their historical memories for the location and quality of food sources. They also exhibit these special traits which are:
	• They can control the recent status of food sources discovered.
	• It can be a reactivated forager by using the information from waggle dance. If other bees confirm the quality of same food source, the same discovered food source will be explored.
	• If the food source is decreasing, scout bees will search new patches.
	• It can be a recruit bee, which is searching a new food source declared in dancing area by another employed bee.

Source: Adapted from Baykasoglu, Ozbakir and Tapkan [21] and Teodorovic, Davidovic and Selmic [22]

According to Teodorovic, Davidovic and Selmic [22], new node will be analysed in the BCO algorithm and added to the partial TSP tour identified in every single step (Table 2). This process is done by a random manner with a certain probabilities. In backward pass process, each bee will decide whether to abandon the partial solution that is generated or keep it. The bees will expand the previous generated partial solution after the selection has been made by a predefined number of nodes during next forward pass, followed by the second backward pass and return to the hive. The decision process will be repeated until complete solution is obtained.

**Table 2:** Bee Colony Optimization algorithm.

Step	BCO Algorithm
1	Initialization: An empty solution is assigned to every bee
2	For every bee // the forward pass:
	Set k = 1 // counter for constructive moves in the forward pass
	Evaluate all possible constructive moves
	According to evaluation, choose one move in using the roulette wheel
3	All bees are back to the hive // backward pass starts
4	Evaluate (partial) objective function value for each bee
5	Every bee decides randomly to continue its own exploration and become a recruiter or become a follower
6	For every follower, choose a new solution from recruiter by the roulette wheel
7	If solutions are not completed, Go to Step 2
8	Evaluate all solutions and find the best one
9	If the stopping condition is not met, Go to Step 2
10	Output the best solution found

Source: Teodorovic, Davidovic and Selmic [22]

In recent studies conducted by Nikolic and Teodorovic [23], they highlighted that in order to design an effective transit network, several issues need to be solved in order to increase number of satisfied riders and at the same time reduce the total time to complete a tour. The optimal solution of transit network design issue is difficult to find which makes it falls under the class of hard combinatorial optimization problem and difficult to be solved without a proper method applied. Therefore, Eq 1 was introduced in the study to calculate the total travel time of all passengers in the network,

T=TT+TW+TTR (1)

where:

TT–total in-vehicle time of all served passengers.

TW–total waiting time of all served passengers.

TTR–total time penalties for all passengers’ transfers (usually time penalty is equal to 5 min per transfer).

Eq 2 is used to calculate the bee’s partial solutions as described in the BCO model:

$$\begin{array}{l} Ob=\underline{T_{max}–T_{b}}\;,b=1,2,\ldots ,B\\ \;T_{max}–T_{min} \end{array}$$ (2)

Where Tb denotes the total travel time generated by the b^th^ bee while Ob denotes the normalized value of the total travel time. T_min_ and T_max_ are the smallest and largest total travel time in the transit networks generated by all bees. To calculate the loyalty of the bee to the previous solution that is generated, Eq (3) is used.

$$Pb=e^{-(Omax-Ob)},b=1,2,\ldots ,B$$ (3)

Omax indicates the maximal normalized value of all bees (Ob). By using this formula (3), a bee can decide whether to become a follower or not. The higher Ob value, the higher the probability of the bee to become a follower and become loyal to the generated solution [1].

#### Exact method

Exact methods are the techniques used by researchers before the heuristic method was introduced and can be considered as the traditional method in solving TSP. Some of the exact methods that are widely used to solve TSP are brute force, dynamic and linear programming [10]. A recent study by Sahalot and Shrimali [24] found that brute force method is a common method used when developing a solution to solve TSP related cases. The brute force method is basically made up of processes that generate all possible tours and calculate every tours distance. The best tour will be the one that with shortest tour identified by using mathematical method (Table 3).

**Table 3:** Brute force processes to solve TSP.

Step	Brute force processes
1	Calculate total possible number of tours by (n-1)! / 2, n represent city
2	List and draw all the possible tours
3	Calculate the distance of each tour
4	Optimal solution is the shortest tour

Source: Sahalot and Shrimali [24]

Awuni [25] identified that brute force approach returned best and most accurate solution all the time, but it is only worked to problems that involve less than 10 cities. Typically, a computer can compute all possible path and distances in a couple of second if the cities are less than 10 where up to 3,628,800 possible routes will be analysed. If only the problem add one more city, the number of possible route will rise by 1000% and this will increase the server load significantly, which is not feasible to be implemented in super computer. Hence, heuristic methods such as bee, ant and genetic algorithms are used to generate the best possible solutions.

## Method

The algorithm is designed to address the prevalent issues of choosing the best route to multiple destinations via RS before going back to the starting point, which can be considered as a variant of TSP. Since exact method capable of generating very reliable solutions in RTSP, solutions generated by using brute force and constraints based on Eq (1) are used as a benchmark for verification purposes. 10 test cases are used to evaluate and compare the solutions generated by the algorithm to the exact solutions. Brute force method is used to search all possible routes that can reach the desired stations before proposing an optimum route at the end of the analysis. This method is slow but accurate in getting the best optimum route to the stations desired provided enough time is given to analyse all paths in the network. In terms of algorithmic complexity, this method is easy to implement but it will be very time consuming depending on the complexity of the RS design.

Thorough observation survey has been conducted to obtain the real travel time to every station in Malaysia RS for algorithm verification usage [26]. The observation approach includes the process of timing and recording the time taken from one station to the next station in minute. To increase the reliability of the data obtained from the observation approach, two observers have been assigned to perform the observation. The first observer controlled the stopwatch and the other observer recorded the time observed. To ensure the data has the consistency, the observers have gone to each station 3 times during different hour of the day to verify the data collected. Variables defined in this survey are travel time from one station to the next closest stations, transit lines, operators, stations name and type. Besides, part of the data required in the verification process is obtained and verified with information obtained from Myrapid official web site (http://www.myrapid.com.my) and google map tool (https://www.google.com/maps).

Data obtained is used to generate 10 RTSP test cases with different settings to examine the reliability of the solutions generated by the algorithm. In the test cases, we have assumed that a salesman has to plan his tour so he manages to attend multiple meetings in Klang Valley by using RS starting from the station nearest to his office and then returning back to the same station at the end of the tour. To avoid analysis error and minimize bias, a simple PHP random function has been used to randomly pick the desired stations included in the tour (Table 4). There are 3 levels of complexity defined in the cases created. 5 cases involve 4 stations, 2 cases with 5 stations and another 3 with 6 stations in the tour. The same PHP function has been used to select initial station in all cases to avoid bias in the research.

**Table 4:** Test cases created to verify the algorithm.

Case	Route	Complexity (stations)
**1**	Kajang → Serdang → Mid Valley → KL Sentral → Kajang	4
**2**	Taman Jaya → KLCC → Bandaraya → Bank Negara → Taman Jaya	4
**3**	Mid Valley → Bandar Tun Razak → Chow Sow Lin → Serdang → Mid Valley	4
**4**	Jalan Templer → Petaling → Pasar Seni → Salak Selatan → Jalan Templer	4
**5**	Abdullah Hukum → Bangsar → Mid Valley → Plaza Rakyat → PWTC → KLCC → Abdullah Hukum	4
**6**	Sungai Buloh → Kepong → Mid Valley → Serdang → Bukit Jalil → Sungai Buloh	5
**7**	Segambut → Bank Negara → Mid Valley → Cheras → UKM → Segambut	5
**8**	Plaza Rakyat → Dang Wangi → Kampung Baru → Pasar Seni → Kuala Lumpur → PWTC → Plaza Rakyat	6
**9**	Raja Chulan → Plaza Rakyat → Bandaraya → Wangsa Maju → Kuala Lumpur → Angkasapuri → Taman Jaya → Raja Chulan	6
**10**	Ampang Park → Wangsa Maju → Dang Wangi → Bandaraya → Kuala Lumpur → Kerinchi → Ampang Park	6

The solutions obtained from both exact and heuristic methods were compared to ascertain the accuracy and reliability of the output. This verification approach is limited by the fact that obtaining exact solutions for complex RS is not likely but still, this approach will help as sanity check for algorithm proposed [27].

## Results- Proposed Novel Bee Inspired Algorithm

**Fig 2:**
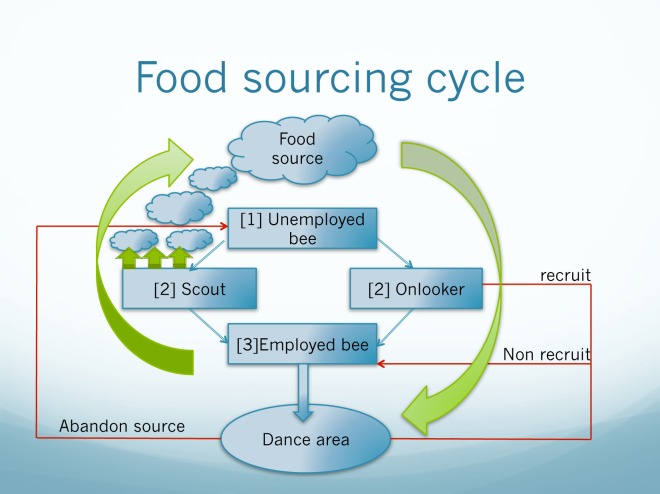
Bee foraging concept used in the algorithm (Table 4) (Source: Adapted from Teodorovic, Davidovic and Selmic [22]).

Fig 2 shows the conceptual view of the solution and the algorithm designed is presented in Table 5. Mathematical model to present the five constraints considered in finding the optimum route (Table 6).

**Table 5:** Proposed bee algorithm to solve RTSP.

**Parameters:**
S as starting point,
G as desired destinations,
T[] as total travelling time,
G[] as temporary storage of G found and analysed,
n as number of possible route from S or IC,
m as number of possible route to identified G,
Z as number of G in the tour,
r as possible route to G from S or IC
**[Unemployed bee] [1]**
The following steps will be repeated for Z times (i = 0; i≤Z)
**[Scout] [2]**
Check line and station type of S
If (IC) Check number of possible route, n
If(n>6){Explore partial available routes for G} else {Explore all r}
**[Employed bee] –share knowledge, backward and forward passes [3]**
If(G found from r){check how many G found and how many r identified that can reach any G, m -> CA1} else {transit to nearest IC and set IC as S}
Check G in the r connecting S
if (G located) {Return to S and check any nearer G via IC in between -> M1} else {Store travelling time, T[], set G as new S and store in G[]}
End
Comparison and analysis 1 (CA1), Calculate time difference to G, T via different routes, Ts
Calculate difference number of station passed before reaching G via 2 different routes, Ns
If Ns>Ts Add 1 minute per station passed as stopping time to T as new temporary time for comparison, t
Select the r with the shortest t, Store travelling time, T[], set G as new S and store in G[]
Else Select r with the shortest T from S to IC to G, denote as T, Store travelling time, T[], set G as new S and store in G[]
End
**[Onlooker] [2]**
if(Z times (i> = Z-1){Display G[]}else{Keep exploring till located all G -> B1}

Note: The indicators [1], [2] and [3] are based on the Fig 2. Bee foraging concept used in the algorithm.

**Table 6:** Overview of the mathematical model with 5 constraints.

**Parameters:**		
m: total number of station		
n: number of routes		
S: starting point or end station		
G: temporary stop or destination		
T_SG_: travel time from node S to node G		
X_SG_: identified optimum route set value to 1		
*T*_*w*_: transit time from one station to another in the interchange		
P_i_: Number of possible route to destination		
L: set of total number routes, L = N		
L’: set of number of possible routes that had been analysed based on probability, P_i_ result		
L′_k_: Optimum path identified		
k: iteration number of collection station name		
i: number of possible routes in the set L’		
N: total number of routes		
Min	$Z=\sum_{S=1}^{m}\sum_{G=1}^{m}T_{SG}X_{SG}$, *where* S ≠ *G*	**(E1)**
Subject to	$P_{i}=\left\{ \begin{array}{l} number\_of\_routes,n<6\\ 0.5*\;number\_o\_route,n\geq 6 \end{array}\right.$	**(E2)**
	$T_{SG}=\left\{ \begin{array}{l} T_{SG}+T_{w},if\_walking\_time\geq 1\;\min\\ T_{SG},otherwise \end{array}\right.$	**(E3)**
*Where*	*T*_*w*_ *is walking time from one station to another station in the interchange*	
	L′ = min{(X_1_,X_2_,….,X_n_)|X_i_ ∈ L, ∀ 1 ≤ i ≤ n}	**(E4)**
*Where*	*i = 1*, *2*,*…*,*n*	
	L′_k_ = X_i_ = T_SG_	
*Where*	*k represent as number of collection station names*	
	$T_{SG}=\left\{ \begin{array}{l} T_{SG}+1,\;if\;more\;than\;1\;route\;can\;lead\;to\;any\;G\\ T_{SG},\;otherwise \end{array}\right.$	**(E5)**
	$X_{SG}=\left\{ \begin{array}{l} 1,\;if\;travel\;time\;is\;included\;in\;the\;route\\ 0,\;otherwise \end{array}\right.$	

There are three major groups of bees in the bee foraging model referred. The groups are the employed bees, onlookers and scouts group [28]. These bees have their own tasks to find nectar around the hive. The information of the food source around the hive gathered by the employed bee will be shared with the onlooker bees. The onlooker bees will evaluate this information to start a neighbourhood search by using a probabilistic approach while a scout bee will perform a random search in order to find a food source [29]. The bees shared the information about their food source by performing a dance, known as the “waggle dance”. One study by Chauhan and Butani [30], shows that the onlooker bee will evaluate the information gathered from the employed bees and select the food source with a greater nectar amount. After that, the bees will memorize the new position of a higher nectar food source and forget or abandon the old one. Solution to RTSP can be easily obtained by using the novel optimization algorithm (Table 5) and mathematical model (Table 6) designed based on the bee foraging concept (Fig 2).

The objective function (E1) is used to find optimum route that have minimum traveling time to multiple destination before returning to the first station. Total tour traveling time is summation of T_SG_ and X_SG_, where SG represents as node S to node G. This summation repeats until m, where m represents number of station to visit.

There are the 5 constraints to be considered in the mathematical model presented (Table 6):

### First Constraint (E1)

$$P_{i}=\left\{ \begin{array}{l} \;number\;of\;routes\;,\;n\;,N<6\;(a)\\ 0.5\;*\;number\;of\;routes\;,n\;,N\geq 6\;(b) \end{array}\right.$$

The first constraint is to check if the number of routes is less than 6, probability (a) will be used. All the routes will be considered and the travel time for each route will be calculated. However, if there are more or equal than 6 routes, probability (b) will be used where only 50 percent of the potential routes will be analyzed. After determining the number of routes to be analyzed, constraints (E2) will be investigated. Number of possible routes is denoted as and number of routes that will be assigned to put into the set L.

### Second Constraint (E2)

$$T_{SG}=\left\{ \begin{array}{l} T_{SG}+T_{w}\;,if\;transiting\;to\;other\;line\geq 1\;minute\\ T_{SG}\;,otherwise \end{array}\right.$$

T_w_ is transiting time from one station to another in different line via the interchange

The second constraint (E2) is used to check whether transiting from node S to node G consumes any time. If the transit consume more than or equal to 1 minute is satisfied, then extra time will be added to the travel time from previous station to this interchange station

### Third Constraint (E3)

$$T_{SG}=\left\{ \begin{array}{l} \;T_{SG}\;+1\;minute\;to\;every\;station\;passed,\;if\;more\;than\;1\;route\;can\;lead\;to\;any\;G\\ T_{SG},\;otherwise \end{array}\right.$$

The third constraint (E3) is to identify whether more than 1 route can lead to any G is identified. If condition more than 1 route that can lead to any G is satisfied, then extra 1 minute will be added for each station passed between nodes S to G. If the condition is not satisfied, station count in between 2 stations will be ignored. If the travel time for identified routes connected to G is same, optimum route will be selected randomly.

### Fourth Constraint (E4)

$$L^{\prime }=min\left\{(X_{1,}X_{2,}\ldots .,X_{n}\right)|X_{i}\in L,\;\forall \;1\leq i\leq n\}$$

Where i = 1, 2,…, n

i represent as number of each possible route that had been choose by using first constraint (E1)

The fourth constraint (E4) is used to select the route with the shortest travelling time among routes connected to the station. In the container, set L’ is known as numbers of possible routes and the route with minimum traveling time to be chosen.

### Fifth Constraint (E5)

$$X_{SG}=\left\{ \begin{array}{l} 1,\;if\;travel\;time\;between\;node\;Sto\;G\;is\;included\;in\;the\;route\\ 0,\;otherwise \end{array}\right.$$

The last constraint is to decide the route and the travel time between node S to node G to be included in the optimum solution after potential routes comparison has been done by using third constraint (E3). If condition is satisfied and the route is chosen, then *X*_*SG*_ is equal to 1.

## Computational Results

The computational study evaluates the robustness of the algorithm through test case and numerical case study. In test case section, the algorithm is compared to the optimum route identified using exact method. In the following section, a representative application of the algorithm developed compared with exact and greedy methods generated from a TSP solver is presented via a numerical case study.

### Test Case

**Fig 3:**
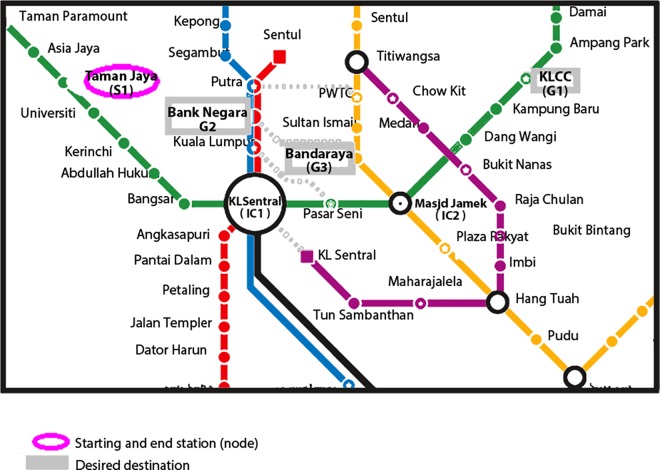
Map of case 2 desired stations in the network.

In this test case, a salesman is required to attend multiple meetings at different locations, Bank Negara, Bandar Raya and KLCC via RS before returning to his office at Taman Jaya (Fig 3). The proposed algorithm has been used to identify the optimum route to the stations required before returning to first station. Table 7 demonstrated how the algorithm is applied to obtain the optimum route of the tour without using any computers.

**Table 7:** Steps to identify the optimum route by using the algorithm.

**Optimum route identification**	**Steps taken using algorithm**
Overview of the tour in the test case	■ Starting point, S1 = Taman Jaya,
	■ Desired destinations, G = {Bank Negara, Bandaraya, KLCC},
	■ T [] as travel time,
	■ [] represents as an array,
	■ G [] as temporary storage of G found and analyse
**Viable routes to next desired station(s)**	
Starting point, S1 = Taman Jaya	■ S1 is not interchange. 2 possible routes identified, r, from Taman Jaya.
	■ Desired destination (G1), KLCC, is found on the same line, *r*_1_
	■ *r*_2_ is abandoned because none G is found.
	■ Travel time from S1 to G1 is stored T[], T1 = 1036 seconds
	■ Interchange KL Sentral (IC1) is found before G1. Expand the search to locate possible G from other connected lines to IC1.
There are 5 alternative routes identified, m	■ 5 bees are sent to explore all routes since the possible route count is less than 6.
from KL Sentral (IC1)	■ G2 is found on *m*_1_ and *m*_2_.
	■ G2 is located, Bank Negara
	■ Number of station from IC1 to G2 is N*m*_2_ = 1
	■ Number of station from IC1 to G1, is N*r*_1_ = 4
	■ Analyze whether station count will affect the results.
	■ Temporary travel time from IC1 to G2 is T*m*_2_ = 4
	■ Temporary travel time from IC1 to G1 is T*r*_1_ = 8
	■ Difference travel time for *m*_1_ and *r*_1_, Ts = T*m*_2_—T*r*_1_ = 4
	■ Difference for *m*_1_ and *r*_1_, Ns = N*m*_2_–N*r*_1_ = 3
	■ Ns is larger than Ts, hence station count is not considered.
	■ Total travel time from Taman Jaya to KL Sentral then Bank Negara via *m*_1_, T2 = 1109 seconds
	■ Compare T1 and T2
	■ T1 has lower travel time. Select previous solution., *r*_1_
	■ KLCC station is stored in G [] and set as new starting point, S2.
Iteration 2 to location 2^nd^ station	■ Starting point, S2 = KLCC
(S2 = KLCC)	■ KLCC is not interchange. 2 routes from stations identified, r from KLCC.
	■ No desired destination found on both routes.
	■ Locate nearest interchange to transit to other line. Interchange (IC2) Masjid Jamek is the nearest IC found in *r*_1_.
	■ Abandon *r*_2_.
	■ Set nearest IC2 as temporary S
	■ Find alternative routes to any G from Masjid Jamek (IC2).
3 routes identified from IC2	■ Desired destination, Bandaraya, G3, is found in route *m*_2_.
	■ Alternative route *m*_1_ and *m*_3_ are abandoned because no desired stations on the routes.
	■ Total travel time from KLCC to Masjid Jamek followed by Bandaraya), T2 = 332 seconds
	■ Bandaraya is stored in G [] and set as a new starting point, S3.
Iteration 2 to locate 3^rd^ station	■ Starting point, S3 = Bandaraya
(S3 = Bandaraya)	■ S3 is not interchange. 2 new possible routes from S3 identified
	■ No G is found on all possible routes. Locate nearest interchange to transit to other lines.
	■ Set nearest Interchanges Masjid Jamek (IC2) as temporary S. Titiwangsa (IC3) is NOT considered because IC2 is nearer to the S.
No desired destination found from interchange Masjid Jamek, IC2.	■ Proceed to locate next nearest interchange from IC2. Interchange KL Sentral, IC1 is found.
	■ 5 routes identified from KL Sentral.
	■ G2, Bank Negara is found on *m*_1_ and *m*_2_.
	■ Travel time from IC to G2 is 480 seconds.
	■ Analyze whether station count in between stations will affect the results.
	■ Both lines are having the same temporary travel time from IC1 to G2 Ts = 4 minutes and number of station count, Ns = 1.
	■ Ns and Ts = 0. Hence, station count can be ignored.
	■ Time travel from Bandaraya–KL Sentral–Bank Negara via *m*_1_, T3 = 795 seconds
	■ Time travel from Bandaraya–KL Sentral–Bank Negara via *m*_2_, T4 = 795 seconds
	■ Both travel time and number of station in between are the same. A route will be randomly chosen.
	■ Bank Negara is stored in G [] and set as new S, S4
Returning to starting point S1	■ Starting point, S4 = Bank Negara
	■ Locate any connected lines that can transit directly to Taman Jay, S1.
	■ None direct route found from Bank Negara to Taman Jaya.
	■ Locate the nearest interchange, KL Sentral, IC1.
	■ There are 6 new possible routes, r, from KL Sentral, IC1.
	■ Search all possible routes that can connect to Taman Jaya line.
	■ S1 found and can be reached via *r*_1_.
	■ Abandon *r*_2_, *r*_3_, *r*_4_, *r*_5_, *r*_6_.
	■ Time travel from Bank Negara to KL Sentral then Taman Jaya, T4 = 1049 seconds
**Display the G[] and total tour travel time:**	G[] = Taman Jaya–> KLCC–> Bandaraya–> Bank Negara–> Taman Jaya
	T[] = T1 + T2 + T3 + T4 = 1036 + 332 + 795 + 1049 = 3212

Table 8 compares the results obtained from the algorithm and the brute force methods. It can be seen from the Table 8 that the result generated by the algorithm matched the optimum route identified using exact method.

**Table 8:** Comparison of solutions between the proposed algorithm and exact method.

	Proposed Algorithm	Exact method 1	Exact method 2	Exact method 3	Exact method 4
**Route**	Taman Jaya	Taman Jaya	Taman Jaya	Taman Jaya	Taman Jaya
	→ KLCC	→ KLCC	→ Bandaraya	→ KLCC	→ KLCC
	→ Bandaraya	→ Bandaraya	→ *Titiwangsa*	→ Bank Negara	→ Bandaraya
	→ Bank Negara	→ Bank Negara	→ *Hang Tuah*	→ Bandaraya	→ *Titiwangsa*
	→ Taman Jaya	→ Taman Jaya	→ *KL Sentral*	→ Taman Jaya	→ *Hang Tuah*
			→ Bank Negara		→ *KL Sentral*
			→ KLCC		→ *Bank* Negara
			→Taman Jaya		→ Taman Jaya
**Travel time (seconds)**	3212	3212	4625	3662	4175
**Stop station(s)**	26	26	40	30	36
**Transfer station(s)**	4	4	4	4	4

Comparing the results from Table 9 below, it shows 9 out of the 10 solutions obtained from the test cases matched the exact solutions. With only one case (case 7) not matching the exact solution’s result, these results suggested that algorithm developed has the potential to perform better and generate reliable results as exact method when it is applied practically to solve TSP. This also further support the idea of using bee intelligence to solve complex problems can be productive.

**Table 9:** Comparison of 10 test cases solutions generated by using exact and proposed algorithm.

Case	Route	Remarks
**1**	Kajang → Serdang → Mid Valley → KL Sentral → Kajang	Similar to exact solution
**2**	Taman Jaya → KLCC → Bandaraya → Bank Negara → Taman Jaya	Similar to exact solution
**3**	Segambut → Bank Negara → Mid Valley → Cheras → UKM → Segambut	Similar to exact solution
**4**	Jalan Templer → Petaling → Pasar Seni → Salak Selatan → Jalan Templer	Similar to exact solution
**5**	Abdullah Hukum → Bangsar → Mid Valley → Plaza Rakyat → PWTC → KLCC → Abdullah Hukum	Similar to exact solution
**6**	Sungai Buloh → Kepong → Mid Valley → Serdang → Bukit Jalil → Sungai Buloh	Similar to exact solution
**7**	Mid Valley → Bandar Tun Razak → Chow Sow Lin → Serdang → Mid Valley	Different optimum route
**8**	Plaza Rakyat → Dang Wangi → Kampung Baru → Pasar Seni → Kuala Lumpur → PWTC → Plaza Rakyat	Similar to exact solution
**9**	Raja Chulan → Plaza Rakyat → Bandaraya → Wangsa Maju → Kuala Lumpur → Angkasapuri → Taman Jaya → Raja Chulan	Similar to exact solution
**10**	Ampang Park → Wangsa Maju → Dang Wangi → Bandaraya → Kuala Lumpur → Kerinchi → Ampang Park	Similar to exact solution

These findings could not be extrapolated to all RS in the world due to various constraints that will affect the reliability of the solutions. For instance, time required to transit to another line, stopping and waiting time in each station might is different if it is controlled by human, congestion of the station and train schedules. However, the tests were successful as it demonstrated that the effectiveness of the algorithm in solving the problem discussed. Further studies, which take these variables into account, will need to be undertaken to enhance the algorithm so it can be applied in different RS without much modification.

The research introduced a new algorithm and method that can be beneficial to business practitioners in enhancing the supply chain and RS transportation users who travel in complex network with hundreds of stations and interchanges such as RS in London, New York, China, Japan, India and Germany. Besides of serving as a future reference on the subject of swarm and collective intelligence in transportation planning and scheduling, the potential of using swarm intelligence in solving complex approximation and routing problems is uncovered. The research demonstrated that bee concept works effectively in RS route planning and could be groundbreaking approaches that will change the way people solve TSP and other operations science problems related to rail freight.

### Numerical Case Study

In order to test the effectiveness of the algorithm in solving TSP, we have used a TSP solver [31] to create 100 cases with different number of vertices and then compared the solutions with the one generated by using the proposed algorithm. In this paper, we present one example taken from the 100 cases known as case 1 to show how the comparison is done among exact method, greedy method and our proposed algorithm.

**Fig 4:**
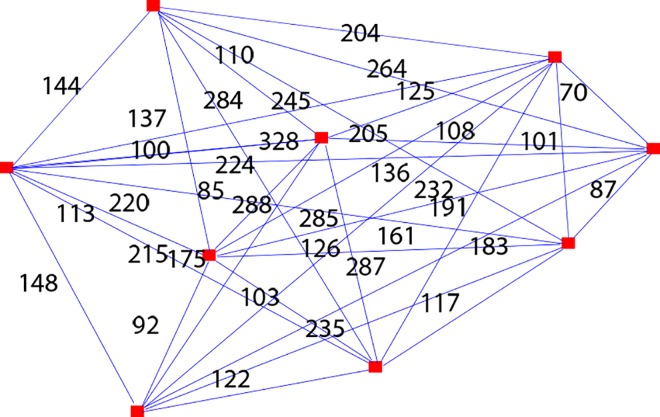
Case 1- Graph with 9 vertices in TSP solver.

Due to practicality and time complexity issues in generating TSP exact solutions with high number of vertices, the solver only allows up to 9 vertices in a graph (Fig 4). Awuni [25] claims the same in his TSP research paper where the brute force algorithm has to perm 10! to compare all routes before returning the solution and the number increases 1000% if an additional vertex is added into a graph. The main objective is not to beat the current best optimization algorithm in solving TSP but to examine the capability of the algorithm in solving TSP when all the constraints proposed are eliminated.

**Fig 5:**
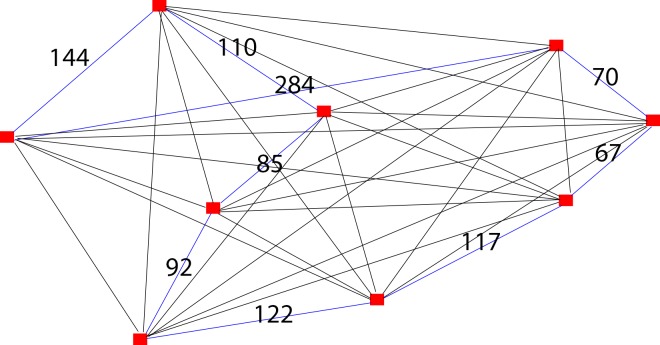
TSP solver solution for case 1 using greedy algorithm (Heuristics methods).

**Fig 6:**
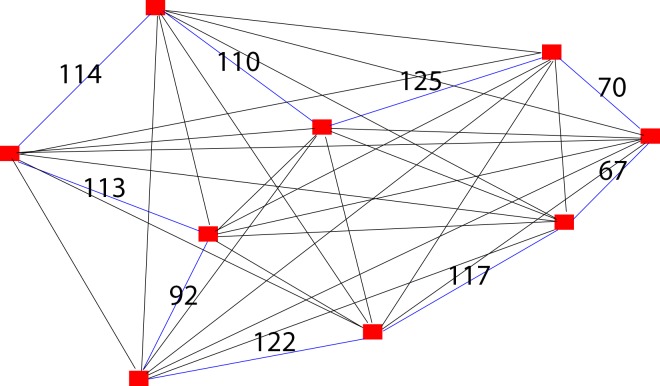
TSP solver solution for case 1 using Brute-Force algorithm (Exact methods).

The solutions generated by the TSP solver for the graph with 9 vertices are shown in Fig 5 (for greedy algorithm or heuristics methods) and Fig 6 (for Brute-Force algorithm or exact methods).

**Fig 7:**
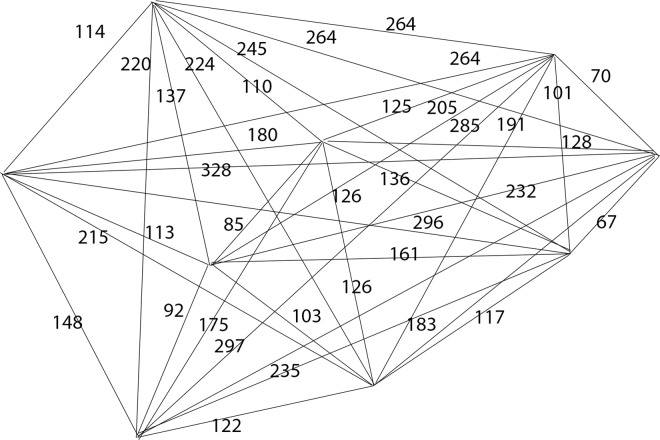
Case 1 routes (redrawn).

Solving case 1 using proposed algorithm first requires consideration of constraints proposed in the algorithm. Since there is no interchange involved, thus the constraints proposed in the algorithm are not applicable in solving TSP. We have eliminated all constraints used in the algorithm so that it is in comparable term with TSP solver solutions (Fig 5 and Fig 6) to test how efficient is our proposed algorithm in solving TSP. Fig 7 is a redrawn diagram of case 1.

**Fig 8:**
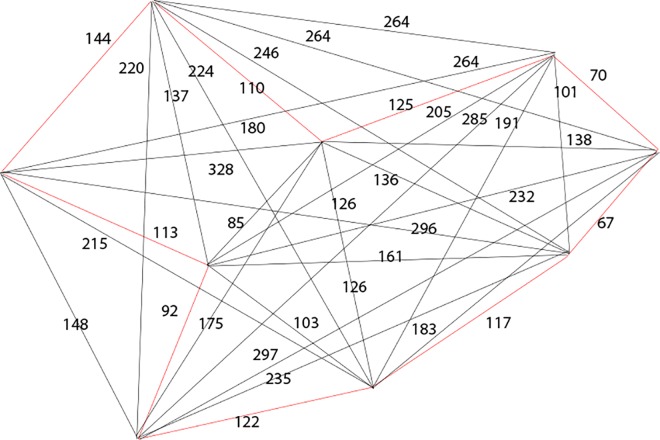
Solution using proposed algorithm.

The proposed bee algorithm to solve case 1 is shown in Table 10. The process 1 to 18 of the proposed bee algorithm can be found in S1 Appendix together with the description of each process. The solution by using proposed algorithm is portrayed in Fig 8 and the comparison of case 1 results generated by exact methods, greedy methods and proposed algorithms is presented in Table 11.

**Table 10:** Proposed bee algorithm to solve case 1.

**Parameters:**
Starting Point, S1 = a
Desired destinations, G = b, c, d, e, f, h, i, j
T[] as total travel distance where[] represents as an array
G[] as temporary storage of G found and analyzed.
n as number of route from S or IC
p as number of possible route to identified G
Z as number of G in the tour
r as m as number of possible route to G from S or IC
**Process [1]: Initialization**
Case 1 consists of 8 desired destinations, G with s as starting and ending point.
**Process [2] Initialize i = 0**
Initialization of the looping process is needed to ensure all destinations are reached before going back to the starting point.
**Process [3] Check i < Z is true**
Number of G in the tour is 8, thus Z = 8
Since i = 0, is less than Z = 8, this condition is true.
**Process [14]:**
Referring to the diagram bee can move to the entire route, thus the number of possible route, n, is 8
n > 6, hence pick randomly possible routes
**Process [15]: Calculate travel distance,**
i. Travel time from a to b = 204
ii. Travel time from a to e = 224
iii. Travel time from a to f = 220
iv. Travel time from a to h = 114
v. Travel time from a to i = 137
vi. Travel time from a to j = 110
**Process [17]:**
Choose the shortest travel distance and set as new starting point S
j is set as new starting point, S2 with 110
**Repeat**
**i = i+1**
Number of G in the tour is 8, thus Z = 8
i = 0+1 = 1 is less than Z = 8
Condition will return true.
Referring to the diagram, bee can move to 7 possible routes. Hence n = 7
n > 6, thus pick randomly possible routes.
Calculate travel distance
i. Travel time from j to b = 125
ii. Travel time from j to c = 138
iii. Travel time from j to h = 180
iv. Travel time from j to f = 175
Choose the shortest total distance and set as new starting point
b is set as new starting point, S3 with 125
**Repeat**
**i = i+1**
Number of G in the tour is 8, thus Z = 8
i = 1+1 = 2 and it is less than Z = 8
Condition will return true.
Referring to the diagram, bee can move to 6 possible routes. Hence n = 6
n> 6, thus pick randomly the possible routes.
Calculate travel distance
i. Travel time from b to c = 70
ii. Travel time from b to d = 104
iii. Travel time from b to e = 191
iv. Travel time from b to f = 285
Choose the shortest distance and set as new starting point
c is set as new starting point, S4 with 70
**Repeat**
**i = i+1**
Number of G in the tour is 8, thus Z = 8
i = 2+1 = 3 and it is less than Z = 8
Condition will return true.
Referring to the diagram, bee can move to 5 possible routes. Hence n = 5
n < 6, thus calculate all the possible routes.
Calculate travel distance
i. Travel time from c to d = 67
ii. Travel time from c to e = 183
iii. Travel time from c to f = 297
Choose the shortest distance and set as new starting point
d is set as new starting point, S5 with 67
**Repeat**
**i = i+1**
Number of G in the tour is 8, thus Z = 8
i = 3+1 = 4 and it is less than Z = 8
Condition will return true.
Referring to the diagram, bee can move to 4 possible routes. Hence n = 4
n < 4, thus calculate all the possible routes.
Calculate travel distance
i. Travel time from d to e = 117
ii. Travel time from d to f = 235
iii. Travel time from d to h = 161
iv. Travel time from d to i = 288
Choose the shortest distance and set as new starting point
e is set as new starting point, S6 with 117
**Repeat**
**i = i+1**
Number of G in the tour is 8, thus Z = 8
i = 4+1 = 5 and it is less than Z = 8
Condition will return true.
Referring to the diagram, bee can move to 3 possible routes. Hence n = 3
n < 6, thus calculate all possible routes.
Calculate travel distance
i. Travel time from e to f = 122
ii. Travel time from e to h = 215
iii. Travel time from e to i = 126
Choose the shortest distance and set as new starting point
f is set as new starting point, S7 with 122
**Repeat**
**i = i+1**
Number of G in the tour is 8, thus Z = 8
i = 5+1 = 6 and it is less than Z = 8
Condition will return true.
Referring to the diagram, bee can move to 2 possible routes. Hence n = 2
N< 6, thus calculate all possible routes.
Calculate travel distance
i. Travel time from f to h = 148
ii. Travel time from f to i = 92
Choose the shortest distance and set as new starting point
i is set as new starting point, S8 with 92
**Repeat**
**i = i+1**
Number of G in the tour is 8, thus Z = 8
i = 6+1 = 7 and it is less than Z = 8
Condition will return true.
Referring to the diagram, bee can move to 1 possible route. Hence n = 1
n < 6, thus calculate all possible routes.
Calculate travel distance
Travel time from i to h = 113
Choose the shortest distance and set as new starting point
h is set as new starting point, S9 with 113
**Repeat**
**i = i+1**
Number of G in the tour is 8, thus Z = 8
i = 7+1 = 8 and it is equal as Z = 8
Condition will return false. Thus it will go to process return trip, [3.B]
Display list of T and calculate travel distance.
List = a→j→b→c→d→e→f→i→h→a
Total distance = 110+125+70+67+117+122+92+113+114 = 930

**Table 11:** Comparison of case 1 results generated by exact, greedy and proposed algorithm.

Method	Proposed Algorithm	Exact Method	Heuristic Method
**Route**	a	a	a
	→ j	→ j	→ j
	→ b	→ b	→ i
	→ c	→ c	→ f
	→ d	→ d	→ e
	→ e	→ e	→ d
	→ f	→ f	→ c
	→ i	→ i	→ b
	→ h	→ h	→ h
	→ a	→ a	→ a
**Total Distance**	930	930	1061

**Fig 9:**
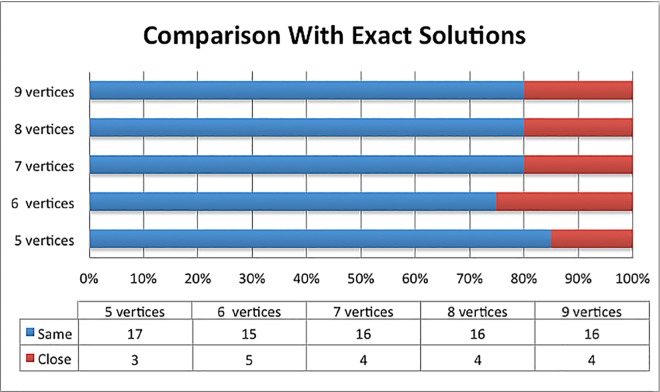
Comparison of proposed algorithm with exact solutions.

All 100 solutions generated by the TSP solver and proposed algorithm in the case study are presented in S2 Appendix. The method proposed managed to generate 80 accurate solutions and the remaining 20 close to the exact solutions. The results obtained from the comparisons are summarized in Fig 9.

Fig 9 shows the results generated by proposed bee algorithm are comparable to the exact solutions with average 80% accuracy. Comparing the results, it can be seen that the increased number of vertices in the cases did not affect the efficiency of the algorithm. These results further support the idea of using the bee inspired algorithm to generate optimal solutions under different environment and constraints.

## Conclusion

Travel planning in the business world is no longer uncommon and often related to one of the most important optimization problem, the Traveling Salesman Problem (TSP). Companies are starting to rely on early planning to achieve the objectives and even tourists can gain wide range of benefits from planning the optimum tour. A novel and verified optimization algorithm and mathematical model based on bee foraging cycle presented in this paper showed how it can be used to solve RTSP, a variant of TSP that received little attention from the researchers efficiently and effectively. This study and analysis also strengthened the idea that the heuristic method used can generate highly reliable solutions. The algorithm can be easily customised and implemented comparing to the exact methods that require higher computational time and resources. The algorithm can be replicated and applied on any RS to solve TSP related cases with different constraints and complexity. Findings of the research can be served as a base for future studies and extend the implication of swarm intelligence in solving TSP, enhancing the supply chain and tour planning. Given the complexity of route planning in cities with hundreds of stations connected in the network such as Tokyo, Seoul, London and New York, there is an opportunity for the use of collective intelligence to enhance the algorithm by using the Knowledge Discovery in Database (KDD) techniques and machine learning theory. Most importantly, the findings help to uncover critical areas in the RTSP that many researchers have not explored and provide opportunity to advance the understanding how swarm intelligence such as bees can be used in route planning.

## Supporting Information

S1 Appendix Flow chart of proposed bee algorithm. (DOCX)

S2 Appendix TSP solutions generated by the TSP solver and proposed algorithm in the experiments conducted. (DOCX)
