# Correction: Bee Inspired Novel Optimization Algorithm and Mathematical Model for Effective and Efficient Route Planning in Railway System

**DOI:** 10.1371/journal.pone.0178583

**Published:** 2017-05-23

**Authors:** The PLOS ONE Staff

The affiliation for the fifth author is incorrect. Siaw-Chuing Loo is not affiliated with #3 but with #4 Centre of Building, Construction & Tropical Architecture, Faculty of Built Environment, University of Malaya, Kuala Lumpur, Malaysia. The publisher apologizes for the error.
