# Supplementary material for: Bee Inspired Novel Optimization Algorithm and Mathematical Model for Effective and Efficient Route Planning in Railway System
Source: PLoS One. 2016 Dec 8;11(12):e0166064. doi: 10.1371/journal.pone.0166064 (PMC5145153; doi:10.1371/journal.pone.0166064)
Supplement: S1 Appendix — (DOCX) [file pone.0166064.s001.docx]

**S1 Appendix: Flow chart of proposed bee algorithm**

**”Yes” denotes as Y, “No” denoted as N*

Start

N

Y

Y

N

N

N

Y

3

3

2

1

[15.B.2] Set new starting point as new S idifference line

[15.B.1] Record travel time, denote as T1

[15.A.3] Spread n bees according to explore better routes G

[15.A.2] Find next G

[15.A.1] Check possible routes for G/multiple G in difference line

[15] IC found between G and S

[14.A] calculated travel time, denote as T1

[14.B] Set nearest IC as new S

[4.B.1] Check if any G in the same line

[14] G found in same line?

[3.B]Display list and travel time

[4.A.1] Check possible routes

[4] S is interchange?

[3.A.2]Check station type of G

[3.A.1] Check S in which line

[2] Initialize i =0

[3] i < Z

[1] Initialize S denoted as starting point and end point, G denoted as desired destination, T [] denote as total travel time, n denoted as number of bees will be sent out, Z denoted as number of G in the tour, m /r denoted as number of possible route to G from S or IC, G [] as temporary storage of G found and analyzed

[15.A.4] Initialize k = 0

2

1

[5] Any possible route

[16] k < n

N

[17] Check any G is found

N

[6] m ≥ 6

N

Y

Y

[17.B] Choose best solution

[17.A]Calculate travel time from IC to G

[6.B] Choose all

[6.A] Choose partial possible routes randomly, except for G identified

[7]Spread n bees to explore better routes according to no. of route

[8]Initialize j = 0

[9] j < n?

Y

N

4

[10.B]Follow the best solution

[10] Any G along the route

Y

5

N

Y

Y

6

[11.A.3] Calculated difference in number of station for routes, denote as Ns

[11.A.1] Check number of station between IC to G / multiple G

[12.A] Convert travel time from second to minute.

[12] Unit in travel time is second?

[11.A] Calculated travel time from IC to G

[11] More than 1 possible route connected to any G / multiple G

[11.B] Calculated travel time, denote as T

4

[11.B.1] Set new starting point as new S

[11.A.2] Calculated difference in travel time for routes, denote as Ts

5

6

[17.A.1] Check number of station from IC to G / multiple G

[17.A.2] Calculated difference in travel time for routes, denote as Ts

[13.1] Record travel time, T

[17.A.3] Calculated difference in number of station for routes, denote as Ns

[13.2] Retrieve travel time and station name

[13.3] Set new starting point as new S

[17.A.6] Set new starting point as new S

[17.A.5] Choose shortest travel time

[17.A.4] Compare T1 and T2

*C2 represent as condition 2. It is a part of the primary flow.

Y

N

[13.A.1] Add 1 minute as train stopping time for each route

[13.B.1] Ignored the train stopping time

[13] Ns > Ts

[13.A.2] Calculated temporary travel time for routes from IC to G, denote as **t**

[13.B.2] Calculated travel time from S to IC to G, denoted as T2

[13.A.3] Compare the temporary travel time

[13.A.4] Choose shortest temporary travel time

[13.A.5] Change the unit in chosen temporary travel time from minute to second

[13.A.6] Calculated travel time (S to IC to G) that had been chosen, denote as T2

*C1 represent as condition 1. It is part of the primary flow.

[18] Need to move to another line

Y

N

[18.A] Add 5 minute as walking time, denote as T_W

[18.B] neglected 5 minute as walking time

**Process & Description**

| **Process** | **Description** |
| --- | --- |
| **1** | initialization S as starting point and ending point, desired destinations will be stored in list of G, station names and travel time will be stored in the list of T, n denotes as the number of bees that will be sent out, total number of desired destinations denote as Z, number of possible route denote a m/r |
| **2** | Initialize i as zero |
| **3** | Repeat until i less than number of desired destination (i < Z) |
| **3.A.1** | Checking line of S |
| **3.A.2** | Checking station type of S |
| **3.B** | When all of the process from [3] is finished, display the list of station that is stored in list T and total time travel calculated |
| **4** | Check whether S is interchange |
| **4.A.1** | If S is interchange, then check number of possible routes, denote as m |
| **4.B.1** | If S is NOT interchange, then check if any G in the same line |
| **5** | Check any possible route |
| **5.B** | If NO any possible route, then check whether need to move to another interchange station |
| **6** | If any possible route, then check whether m is greater than or equal to 6 lines |
| **6.A** | If m ≥ 6 lines, then choose partial possible routes randomly, except for G identified |
| **6.B** | If m < 6, then choose all possible routes |
| **7** | Spread n bees according to explore better routes |
| **8** | Initialize j = 0 |
| **9** | Repeat until j equal to number of bees |
| **10** | Check whether any G along the route |
| **10.B** | If NO any G along the route, then follow the best solution has found earlier. |
| **11** | If any G along the route, then check whether more than 1 possible route connected to any G/multiple G |
| **11.A** | If more than 1 possible route connected to any G / multiple G, then calculated travel time from IC to G |
| **11.B** | If less than 1 possible route connected to any G / multiple G, then calculated travel time, denote as T |
| **11.B.1** | Set new starting point as new S |
| **12** | Check whether unit in travel time is second |
| **12.A** | If unit in travel time is not second, then convert travel time from second to minute. |
| **11.A.1** | Check number of station between IC to G/multiple G |
| **11.A.2** | Calculated difference in travel time for routes, denote as Ts |
| **11.A.3** | Calculated difference in number of station for routes, denote as Ns |
| **13** | Check whether Ns > Ts |
| **13.A.1** | If Ns > Ts, then add 1 minute as train stopping time for each route |
| **13.A.2** | Calculated temporary travel time for routes from IC to G, denote as **t** |
| **13.A.3** | Compare the temporary travel time |
| **13.A.4** | Choose shortest temporary travel time |
| **13.A.5** | Change the unit in chosen temporary travel time from minute to second |
| **13.A.6** | Calculated travel time (S to IC to G) that had been chosen, denote as T2 |
| **13.B.1** | If Ns ≤ Ts, then ignored the train stopping time |
| **13.B.2** | Calculate travel time from S to IC to G, denote as T2 |
| **13.1** | Record travel time, T |
| **13.2** | Retrieve travel time and station name |
| **13.3** | Set new starting point as new S |
| **14** | Check whether G is found in same line |
| **14.A** | If G found in the same line as S, then calculated travel time, denote as T1 |
| **14.B** | If NO G found in the same line as S, then set nearest interchange as temporary S |
| **15** | Check whether IC found between G and S |
| **15.A.1** | If IC found between G and S, then check possible routes for G / multiple G in difference line |
| **15.A.2** | Find next G |
| **15.A.3** | Spread n bees according to explore better routes |
| **15.A.4** | Initialize k = 0 |
| **15.B.1** | If IC is NOT found between G and S , then record travel time, denote as T1 |
| **15.B.2** | Set new starting point as new S |
| **16** | Repeat until k equal to number of possible route |
| **17** | Check any G is found |
| **17.A** | If any G is found, then calculate travel time from IC to G |
| **17.B** | If NO G is found, then choose best solution found that has found |
| **17.A.1** | Check number of station from IC to G/multiple G |
| **17.A.2** | Calculated difference in travel time for routes, denote as Ts |
| **17.A.3** | Calculated difference in number of station for routes, denote as Ns |
| **17.A.4** | Compare T1 and T2 |
| **17.A.5** | Choose shortest travel time |
| **17.A.6** | Set new starting point as new S |
| **18** | Check whether need to move to another line |
| **18.A** | If need to move to another line, then add 5 minute as walking time, denote as T_W |
| **18.B** | If NOT need to move to another line, then neglected 5 minute as walking time |
