# Supplementary material for: Bee Inspired Novel Optimization Algorithm and Mathematical Model for Effective and Efficient Route Planning in Railway System
Source: PLoS One. 2016 Dec 8;11(12):e0166064. doi: 10.1371/journal.pone.0166064 (PMC5145153; doi:10.1371/journal.pone.0166064)
Supplement: S2 Appendix — (DOCX) [file pone.0166064.s002.docx]

**S2 Appendix: TSP solutions generated by the TSP solver and proposed algorithm in the experiments conducted**

| **Cases** | **No. of Vertices** | **Heuritics Method** | **Exact Method** | **Matched/Not Matched** |
| --- | --- | --- | --- | --- |
|  | **5** | **595** | **595** | **Matched** |
|  | **5** | **510** | **510** | **Matched** |
|  | **5** | **483** | **483** | **Matched** |
|  | **5** | **462** | **462** | **Matched** |
|  | **5** | **421** | **421** | **Matched** |
|  | **5** | **558** | **558** | **Matched** |
|  | **5** | **445** | **445** | **Matched** |
|  | **5** | **424** | **424** | **Matched** |
|  | **5** | **689** | **582** | **Not Matched** |
|  | **5** | **480** | **445** | **Not Matched** |
|  | **5** | **498** | **498** | **Matched** |
|  | **5** | **543** | **543** | **Matched** |
|  | **5** | **412** | **412** | **Matched** |
|  | **5** | **465** | **465** | **Matched** |
|  | **5** | **513** | **500** | **Not Matched** |
|  | **5** | **570** | **570** | **Matched** |
|  | **5** | **540** | **540** | **Matched** |
|  | **5** | **528** | **528** | **Matched** |
|  | **5** | **661** | **661** | **Matched** |
|  | **5** | **552** | **552** | **Matched** |
|  | **6** | **701** | **701** | **Matched** |
|  | **6** | **776** | **776** | **Matched** |
|  | **6** | **490** | **423** | **Not Matched** |
|  | **6** | **509** | **509** | **Matched** |
|  | **6** | **692** | **648** | **Not Matched** |
|  | **6** | **557** | **557** | **Matched** |
|  | **6** | **686** | **686** | **Matched** |
|  | **6** | **548** | **507** | **Not Matched** |
|  | **6** | **637** | **637** | **Matched** |
|  | **6** | **606** | **606** | **Matched** |
|  | **6** | **610** | **610** | **Matched** |
|  | **6** | **746** | **612** | **Matched** |
|  | **6** | **648** | **648** | **Matched** |
|  | **6** | **492** | **492** | **Matched** |
|  | **6** | **556** | **499** | **Not Matched** |
|  | **6** | **563** | **563** | **Matched** |
|  | **6** | **715** | **715** | **Matched** |
|  | **6** | **624** | **624** | **Matched** |
|  | **6** | **683** | **682** | **Matched** |
|  | **6** | **518** | **518** | **Matched** |
|  | **7** | **936** | **936** | **Matched** |
|  | **7** | **843** | **843** | **Matched** |
|  | **7** | **837** | **837** | **Matched** |
|  | **7** | **672** | **672** | **Matched** |
|  | **7** | **909** | **800** | **Not Matched** |
|  | **7** | **856** | **856** | **Matched** |
|  | **7** | **751** | **668** | **Not Matched** |
|  | **7** | **669** | **669** | **Matched** |
|  | **7** | **751** | **751** | **Matched** |
|  | **7** | **733** | **733** | **Matched** |
|  | **7** | **701** | **701** | **Matched** |
|  | **7** | **625** | **625** | **Matched** |
|  | **7** | **577** | **577** | **Matched** |
|  | **7** | **548** | **548** | **Matched** |
|  | **7** | **529** | **529** | **Matched** |
|  | **7** | **586** | **586** | **Matched** |
|  | **7** | **729** | **641** | **Not Matched** |
|  | **7** | **469** | **469** | **Matched** |
|  | **7** | **623** | **623** | **Matched** |
|  | **7** | **718** | **630** | **Not Matched** |
|  | **8** | **1040** | **753** | **Not Matched** |
|  | **8** | **860** | **860** | **Matched** |
|  | **8** | **728** | **728** | **Matched** |
|  | **8** | **776** | **710** | **Not Matched** |
|  | **8** | **809** | **809** | **Matched** |
|  | **8** | **1088** | **1088** | **Matched** |
|  | **8** | **925** | **844** | **Not Matched** |
|  | **8** | **785** | **785** | **Matched** |
|  | **8** | **808** | **808** | **Matched** |
|  | **8** | **758** | **758** | **Matched** |
|  | **8** | **652** | **594** | **Not Matched** |
|  | **8** | **719** | **719** | **Matched** |
|  | **8** | **554** | **554** | **Matched** |
|  | **8** | **733** | **733** | **Matched** |
|  | **8** | **958** | **958** | **Matched** |
|  | **8** | **1025** | **877** | **Not Matched** |
|  | **8** | **983** | **983** | **Matched** |
|  | **8** | **196** | **196** | **Matched** |
|  | **8** | **409** | **409** | **Matched** |
|  | **8** | **497** | **497** | **Matched** |
|  | **9** | **658** | **658** | **Matched** |
|  | **9** | **559** | **559** | **Matched** |
|  | **9** | **880** | **768** | **Not Matched** |
|  | **9** | **939** | **849** | **Not Matched** |
|  | **9** | **930** | **930** | **Matched** |
|  | **9** | **972** | **972** | **Matched** |
|  | **9** | **833** | **833** | **Matched** |
|  | **9** | **894** | **894** | **Matched** |
|  | **9** | **979** | **918** | **Not Matched** |
|  | **9** | **772** | **772** | **Matched** |
|  | **9** | **541** | **541** | **Matched** |
|  | **9** | **725** | **725** | **Matched** |
|  | **9** | **716** | **716** | **Matched** |
|  | **9** | **724** | **724** | **Matched** |
|  | **9** | **633** | **618** | **Not Matched** |
|  | **9** | **606** | **606** | **Matched** |
|  | **9** | **766** | **766** | **Matched** |
|  | **9** | **757** | **757** | **Matched** |
|  | **9** | **753** | **753** | **Matched** |
|  | **9** | **766** | **766** | **Matched** |
